# Rapid dark aging of biomass burning as an overlooked source of oxidized organic aerosol

**DOI:** 10.1073/pnas.2010365117

**Published:** 2020-12-14

**Authors:** John K. Kodros, Dimitrios K. Papanastasiou, Marco Paglione, Mauro Masiol, Stefania Squizzato, Kalliopi Florou, Ksakousti Skyllakou, Christos Kaltsonoudis, Athanasios Nenes, Spyros N. Pandis

**Affiliations:** ^a^Institute of Chemical Engineering Sciences, Foundation for Research & Technology-Hellas, Patras 26504, Greece;; ^b^Institute of Atmospheric Sciences and Climate, Italian National Research Council, Bologna 40129, Italy;; ^c^School of Architecture, Civil and Environmental Engineering, Swiss Federal Institute of Technology Lausanne, Lausanne 1015, Switzerland;; ^d^Department of Chemical Engineering, University of Patras, Patras 26504, Greece

**Keywords:** biomass burning, nighttime, secondary organic aerosol, oxidation, air pollution

## Abstract

Oxidized organic aerosol (OOA) is a major component of ambient particulate matter, substantially impacting climate, human health, and ecosystems. OOA is readily produced in the presence of sunlight, and requires days of photooxidation to reach the levels observed in the atmosphere. High concentrations of OOA are thus expected in the summer; however, our current mechanistic understanding fails to explain elevated OOA during wintertime periods of low photochemical activity that coincide with periods of intense biomass burning. As a result, atmospheric models underpredict OOA concentrations by a factor of 3 to 5. Here we show that fresh emissions from biomass burning exposed to NO_2_ and O_3_ (precursors to the NO_3_ radical) rapidly form OOA in the laboratory over a few hours and without any sunlight. The extent of oxidation is sensitive to relative humidity. The resulting OOA chemical composition is consistent with the observed OOA in field studies in major urban areas. Additionally, this dark chemical processing leads to significant enhancements in secondary nitrate aerosol, of which 50 to 60% is estimated to be organic. Simulations that include this understanding of dark chemical processing show that over 70% of organic aerosol from biomass burning is substantially influenced by dark oxidation. This rapid and extensive dark oxidation elevates the importance of nocturnal chemistry and biomass burning as a global source of OOA.

## Significance

To quantify the full implications of biomass burning emissions on the atmosphere, it is essential to accurately represent the emission plume after it has undergone chemical aging in the atmosphere. Atmospheric models typically consider the predominant aging pathway of biomass burning emissions to take place in the presence of sunlight (via the OH radical); however, this mechanism leads to consistent underpredictions of oxidized organic aerosol in wintertime urban areas. Here, we show, through a combination of laboratory experiments, ambient field measurements, and chemical transport modeling, that biomass burning emission plumes exposed to NO_2_ and O_3_ age rapidly without requiring any sunlight, thus providing an overlooked source of oxidized organic aerosol previously not accounted for in models.

Highly oxidized organic aerosol (OOA) is a dominant component of particulate matter air pollution globally (1–3); however, sources of OOA remain uncertain, limiting the ability of models to accurately represent OOA and thus predict the associated climate, ecosystem, and health implications (4, 5). The current conceptual model of OOA formation suggests that anthropogenic OOA predominantly originates from the oxidation of volatile (VOCs), intermediate volatility (IVOCs), and semivolatile (SVOCs) organic compounds by the OH radical, resulting in lower-volatility products that condense to the particle phase (6). As the OH radical is formed through photolysis and has a very short atmospheric lifetime [less than a second (7)], this oxidation mechanism only occurs in the presence of sunlight. Further, the time scale for OOA formation through oxidation with OH in models is on the order of a few days (8). While this understanding is sufficient in explaining OOA concentrations in summer or periods with high solar radiation, atmospheric models fail to reproduce the observed concentration of OOA in the ambient atmosphere during winter and low-light conditions (9, 10). Fountoukis et al. (9) found simulated OOA concentrations significantly underestimated in wintertime Paris. Tsimpidi et al. (10) also reported an underprediction of simulated OOA globally in winter, suggesting missing sources of both primary OA (POA) and secondary formation pathways. This underproduction suggests a possible overlooked conversion pathway of organic vapors or particles to OOA that is not accounted for in current chemical transport and climate models.

As stricter controls on fossil fuel combustion are implemented, residential biomass burning (BB) as a source of heating or cooking is becoming an increasingly important source of OA in urban environments (1, 11, 12). Further, increasing rates of wildfires from climate change are increasing the frequency of smoke-impacted days in urban areas (12–14). BB emissions include high concentrations of POA, SVOCs, IVOCs, and VOCs (15, 16), thus making BB a key source of OOA. Previous research has focused on quantifying the concentration of OOA formed through photochemical oxidation reactions (i.e., OH) with BB emissions (17, 18). However, oxidation of BB emissions in low or no sunlight is less well understood and is not included in chemical transport models. As opposed to OH, the NO_3_ radical is formed through reactions with NO_2_ and O_3_ and is rapidly lost in the presence of sunlight (19). Thus, the NO_3_ radical is only available in significant concentrations at night or other low-light conditions (20, 21). Previous research has established that biogenic VOCs may undergo oxidation at night when mixed with anthropogenic emissions containing NO_2_ and O_3_ (19, 22–27). There have been only a few studies that consider that nighttime oxidation of residential wood combustion may proceed through similar pathways (28–31); however, the magnitude and relevance to observed OOA in the ambient atmosphere has not yet been established. By combining laboratory experiments and ambient observations to inform a chemical transport model, we present strong evidence that nighttime oxidation of BB plumes (proceeding through reactions with O_3_ and the NO_3_ radical) is an important source of OOA.

## Results

### An Overlooked Nighttime Mechanism of Aging in Polluted Environments.

**Fig. 1.**
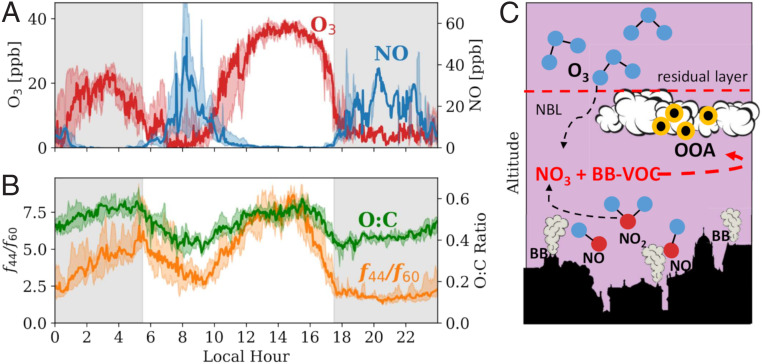
Field observations from an urban center in wintertime Greece show an increasing trend in chemical aging of bbOA at night, roughly correlated with transport of O_3_ into the NBL. Diurnal profiles calculated from 16 d of ambient observations from ambient observations taken in Patras, Greece, on January 14−30, 2020 of (*A*) O_3_ and NO mixing ratios and (*B*) the ratio of oxygen to carbon (O:C) and the ratio of the fraction of OA mass present at *m*/*z* 44 (*f*_44_) to *m*/*z* 60 (*f*_60_). The solid lines represent the median of all measurements taken at the given time of day from the 16-d sampling period. The shaded region around the line represents the 25th and 75th percentiles of measurements across the sampling period. The gray shaded regions represent night. (*C*) Schematic representation of oxidation of bbOA in an urban area due to reactions with the NO_3_ radical produced through O_3_ mixing into the NBL and subsequent reactions with NO_x_.

Formation of NO_3_ at night requires a source of O_3_ to react with NO_2_. Previous studies on nighttime oxidation of biogenic VOCs suggest that the required O_3_ is provided through mixing from the residual layer where O_3_ remains from daytime formation (24–26); however, it is unclear whether this mechanism is important in areas with high BB emissions, as reactions with NO may titrate any transported O_3_. To address this, we analyze 16 d of ambient gas and particle-phase measurements that were recently carried out (January 14–30, 2020) in a wintertime urban center in western Greece (*SI Appendix*, Fig. S1). This site is representative of a typical urban center with a large wintertime BB influence. As expected, residential BB together with transportation emit NO which rapidly consumes any O_3_ remaining in the nocturnal boundary layer (NBL) from daytime formation. This anticorrelation of NO and O_3_ (Fig. 1*A*) is evident as residential BB begins after sunset (17:30 local time [LT]). As the period of fresh emissions of residential BB ends around 21:00 LT, NO is diluted or consumed by reaction with O_3_ mixing slowly down from the residual layer. This O_3_ is then able to achieve substantial concentrations in the NBL, reaching 20 parts per billion (ppb) at 2:00 LT (Fig. 1*A*). This O_3_ can react with NO_2_ to form NO_3_, thus providing an oxidant source for nighttime processing of the primary BB emissions.

To quantify the evolution of OA composition, we follow its oxygen-to-carbon (O:C) ratio and the fractional OA mass signal at *m*/*z* 44 to *m*/*z* 60 (*f*_44_/*f*_60_) measured by a high-resolution time-of-flight aerosol mass spectrometer (HR-ToF-AMS; see *Materials and Methods*). These metrics are commonly used to quantify chemical processing of BB OA (bbOA) in both laboratory and ambient conditions (18). Consistent with the conceptual model of daytime OH-driven oxidation as the dominant source of OOA, diurnal profiles shown in Fig. 1*B* of O:C and *f*_44_/*f*_60_ show a late-afternoon peak indicating processing of OA. However, in contrast to this conceptual model, there is a second peak in O:C and *f*_44_/*f*_60_ at night, which cannot be explained by photolysis-driven oxidation. The nighttime peak in O:C and *f*_44_/*f*_60_ occurs a few hours after a peak in residential BB emissions evidenced by high concentrations of NO (Fig. 1*A*) from 18:00 LT to 20:00 LT. We note that the observations presented here do not show conclusive evidence of nighttime oxidation of bbOA (as other processes such as transport likely also influence the observed profiles of OA evolution); however, these observations are consistent with the mechanism of nighttime oxidation of BB emissions via the NO_3_ radical shown schematically in Fig. 1*C*.

### Rapid Processing of Laboratory BB Emissions under Dark Conditions.

To explore the extent of chemical processing of BB plumes under dark or low-light conditions, we performed environmental chamber experiments with residential BB emissions in the dark exposed to a variety of NO_2_ and O_3_ concentrations (30 ppb to 90 ppb) under dry (around 10%) and high relative humidity (RH; 50 to 80%) conditions (*SI Appendix*, Table S1), as well as reference experiments without the addition of NO_2_ and O_3_ and photolysis-driven-oxidation experiments with ultraviolet (UV) lights.

**Fig. 2.**
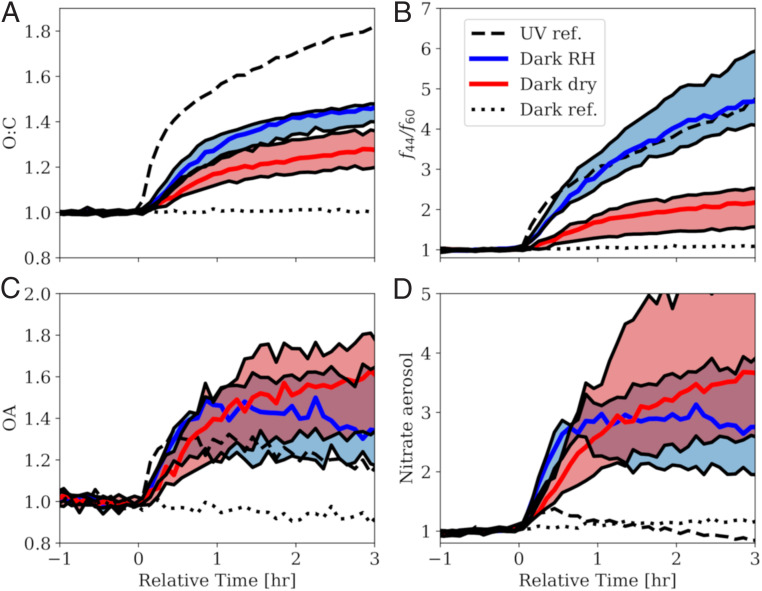
In the presence of NO_2_ and O_3_, BB emissions age rapidly under dark conditions, producing a similar amount of secondary aerosol as under photochemical conditions. Enhancement in (*A*) O:C ratio, (*B*) *f*_44_/*f*_60_, (*C*) OA, and (*D*) total (inorganic and organic) aerosol nitrate for the dark reference (experiment 1 in *SI Appendix*, Table S1) and UV reference (experiment 2) experiments as well as the dark aging experiments under dry (experiments 3 to 6) and high RH (experiments 7 to 9) conditions. The shaded region corresponds to the variability across all experiments (see *SI Appendix*, Table S1) due to differences in injected NO_2_ and O_3_ concentration (dark dry experiments) or RH (dark RH experiments), while the solid blue and red lines are the median across the experiments. Time is relative to the initiation of oxidation (at hour 0). The calculation of enhancement in OA and nitrate aerosol is normalized to sulfate to account for particle wall losses and collection efficiency.

After the initiation of oxidation, the composition of the bbOA evolves dramatically under photolysis-induced oxidation (simulating daytime aging), while, in the experiment with no oxidants or UV lights, the OA mass spectrum remains constant throughout the experiment (Fig. 2 *A* and *B*). This, at a first glance, is consistent with the conceptual model that evolution of bbOA into OOA occurs only in the daytime. However, in the presence of NO_2_ and O_3_, the dark aging experiments under dry and high RH conditions show a noticeable increase (20 to 50% increase over 2 h) in the O:C ratio and a similar or greater enhancement in *f*_44_/*f*_60_ (factor of 1.6 to 6.0) compared to experiments with photooxidation (Fig. 2*B*).

This dark aging of bbOA is sensitive to RH. Dark aging experiments under high RH conditions consistently showed greater enhancement in O:C (1.4 to 1.5) and *f*_44_/*f*_60_ ratios (4.2 to 6.0) relative to dark aging experiments under dry conditions (1.2 to 1.4 for O:C and 1.6 to 2.5 for *f*_44_/*f*_60_), suggesting a sensitivity of the aging process to water vapor and/or aerosol liquid water content. The discrepancy in the enhancement in *f*_44_/*f*_60_ between the dry and high RH experiments is largely caused by a difference in the evolution of the HR-ToF-AMS measured OA signal at *m*/*z* 60 [representing levoglucosan and other anhydrosugars (32); *SI Appendix*, Fig. S2].

The dark aging experiments show a substantial enhancement in OA (20 to 70%) and secondary inorganic and organic aerosol nitrate (factor of 4). The latter accounts for 20 to 30% of the total secondary aerosol formed in these experiments (Fig. 2 *C* and *D*), of which 50 to 60% is organic (*SI Appendix*, Fig. S3). In all experiments, a small amount of organic nitrates (∼0.1 μg⋅m^−3^ to 0.5 μg⋅m^−3^, or 40% of the total aerosol nitrate) is emitted with the initial BB plume. The concentration of organonitrates increases with exposure to NO_2_ and O_3_, suggesting a secondary aerosol formation pathway with the dark oxidation mechanism. The enhancement in aging metrics coincides with decreasing concentrations of measured phenolic (phenols and cresols) and furanoic VOCs (*SI Appendix*, Fig. S4), in good agreement with previous studies (28). The dark oxidation of these VOCs is occurring mostly via reactions with the NO_3_ radical. Experiments without any additional NO_2_ added to the chamber tend to have lower amounts of chemical evolution than those with additional NO_2_ (*SI Appendix*, Fig. S5). These experiments show that, under a variety of oxidant concentrations and RH, bbOA is rapidly oxidized when exposed to NO_3_ radical precursors.

### Evolution of bbOA Spectra into OOA.

**Fig. 3.**
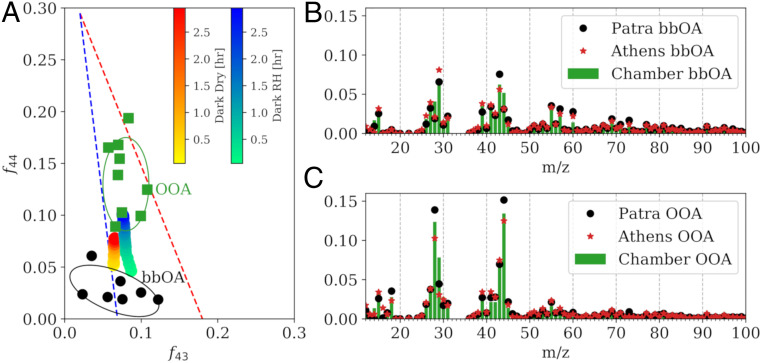
The evolution of OA experimentally under dark conditions compares well to ambient observations of bbOA and OOA factors. (*A*) Oxidation pathway of a representative dark dry (experiment 3) and high RH (experiment 8) experiment compared to ambient observations of bbOA and OOA factors (*SI Appendix*, Tables S2 and S3). (*B*) The OA mass spectra in the dark dry experiment as measured by the HR-ToF-AMS of the fresh bbOA in the chamber compared to the bbOA in Athens and Patras, and (*C*) the produced OOA in the chamber compared to OOA factors in Athens and Patras.

To explore whether the bbOA aged in the dark in laboratory experiments may contribute to ambient OOA in urban centers, we compare the evolution of the HR-ToF-AMS spectra from the chamber experiments to ambient observations. In the triangle plot (33) in Fig. 3*A* depicting the evolution of OA through *m*/*z* 43 and 44 space, the dark aging chamber experiments under dry and high RH conditions are initially representative of ambient bbOA factors observed in wintertime cities impacted by residential BB emissions. After a short time period (3 h) of exposure to NO_2_ and O_3_, the total OA in the chamber experiments evolves to occupy the same space in the triangle plot as the wintertime OOA factors. Similar behavior is also seen in the evolution of OA through *m*/*z* 60 and 44 space (*SI Appendix*, Fig. S6). The OA spectrum evolves rapidly following the initiation of oxidation. The theta angle between the OA spectra (see *Materials and Methods*, which quantifies the degree of dissimilarity between two spectra), after the initiation of oxidation relative to prior oxidation, increases immediately for both the dry and high RH experiments (*SI Appendix*, Fig. S7).

We separate the total OA in the chamber experiments into a fresh bbOA factor and a produced bbOOA (secondary OA [SOA]) factor using a mass balance approach (see *Materials and Methods*). As a whole, the bbOA and bbOOA spectra from the laboratory dark aging experiments show a close agreement with spectra from ambient observations. The theta angle between the produced bbOOA in the dark aging experiment and the ambient OOA factors ranges between 13° and 17° when comparing HR spectra observed in Patras (11) and Athens (11), Greece, Bologna, Italy (34), and Barcelona, Spain (35) (*SI Appendix*, Table S2), and 8° to 17° when comparing unit mass resolution spectra observed in Po Valley, Italy (36), Paris, France (37), and Fresno, CA (38). This suggests relatively good agreement between the mass spectra, and suggests that bbOA aged in dark conditions can contribute to observed OOA concentrations. The bbOOA from the dark aging experiments and ambient OOA factors share prominent peaks commonly associated with oxidized and SOA at *m*/*z* 28, 29, and 44 (32) (Fig. 3*C*). The laboratory-produced bbOOA does not exhibit a high degree (theta angle of 31°) of similarity with the nitrate-related OOA (NOOA) from Fresno, CA, reported in ref. 39. Some of this difference can be attributed to the relatively larger normalized OA signal at *m/z* 16 (CH_4_^+^) and smaller normalized OA signal at *m/z* 28 in ref. 39 compared to the laboratory-produced OOA.

Some of the differences in spectra noted above are at least partially due to differences in the chemical composition of the fresh bbOA. Comparing the spectra of the fresh bbOA from our experiments to the corresponding fresh bbOA spectra in the cities discussed above (*SI Appendix*, Table S3), we find theta angles ranging between 16° and 36° (Fig. 3*B*). In some locations (e.g., Athens, Patras, and Fresno), this suggests reasonable but not perfect agreement between the mass spectra of the fresh bbOA factors, while, for other locations, there are fairly different spectra. The differences in the fresh bbOA factors (*SI Appendix*, Figs. S8 and S9) may be caused by differences in fuel type and combustion conditions or possibly by varying degrees of atmospheric processing (for instance, different dilution rates may cause evaporation of primary particles).

### Dark Oxidation Enhances bbOA to bbOOA Conversion in Models.

**Fig. 4.**
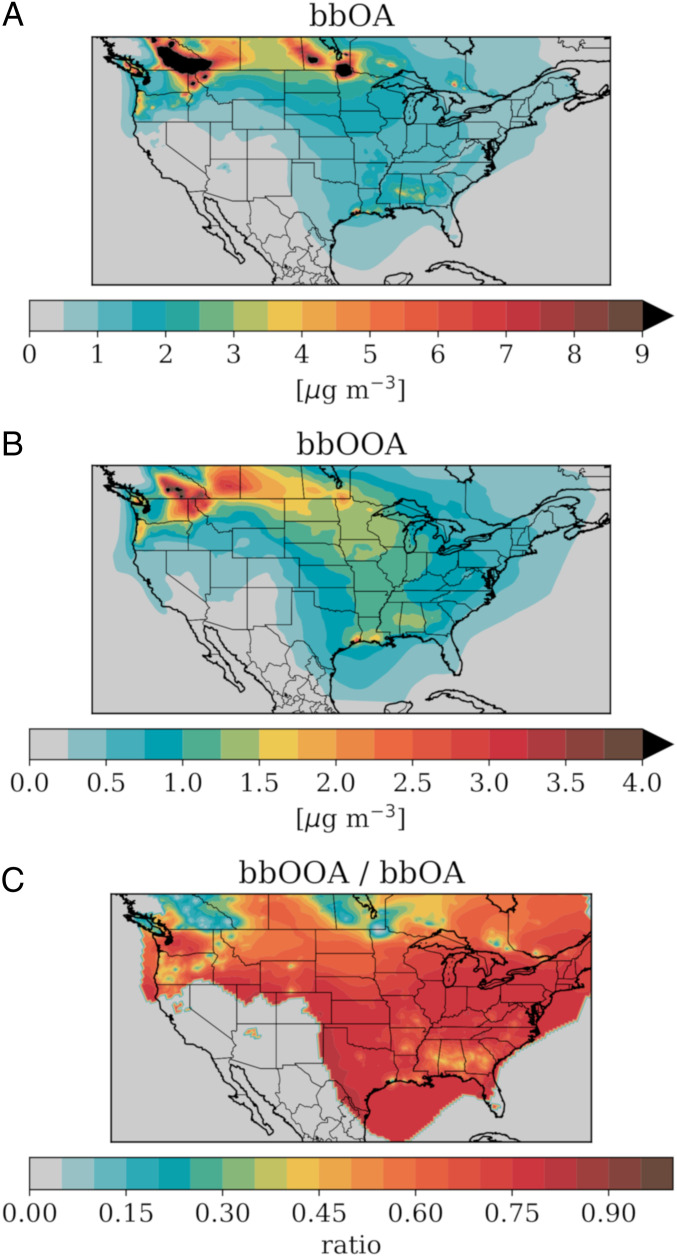
Model simulations predict that more than 75% of total OA from BB sources over the United States is influenced by dark aging. Concentrations of simulated (*A*) total bbOA mass (both primary and secondary) and (*B*) OOA mass from BB sources that has undergone at least one nighttime NO_3_ reaction, as well as (*C*) the ratio of bbOOA mass (where bbOA is greater than 0.5 μg⋅m^−3^) influenced by dark aging to total bbOA.

To quantify the extent that dark aging influences OA emitted from BB sources, we incorporate NO_3_ radical oxidation of gas-phase VOCs from BB emissions into a regional chemical transport model applied over the United States (see *Materials and Methods*) during an autumn period characterized by major wildfires in Canada, Montana, and Idaho (Fig. 4*A*). Downwind of fires over much of the central and eastern United States, the simulated total bbOA had concentrations ranging from 1 μg⋅m^−3^ to 5 μg⋅m^−3^, suggesting that BB sources were an important contributor to background aerosol concentrations during the simulated period.

In the simulation with the NO_3_ radical oxidation included, POA decreased relative to simulations without NO_3_ oxidation by 20 to 30% downwind of active fires, accompanied by an increase in bbOOA (*SI Appendix*, Fig. S10). This increase is most prevalent in active fire regions where bbOOA increased by more than 50 to 60% from dark NO_3_ aging. The inclusion of dark NO_3_ oxidation of bbOA leads to rapid oxidation and a shift from primary to more oxidized OA. Nighttime-influenced bbOOA propagates throughout much of the United States, with maxima in the fire regions of the Pacific Northwest and transport to the central and eastern United States (Fig. 4*B*). The influence of dark oxidation on the total bbOA burden can be estimated from the ratio of the dark-influenced bbOOA to the total bbOA. Using this metric, we find that, in regions where the bbOA burden is greater than 0.5 μg⋅m^**−3**^, up to 60 to 70% of the total OA from BB emissions across the United States during this late fall period is influenced by dark chemical processing. As the smoke plume is transported farther downwind from active fires (such as Southern California and Mexico), almost all of the OA associated with BB has undergone some nocturnal NO_3_ chemistry. In active fire regions with high concentrations of bbPOA, 10 to 15% of the total OA is still influenced by this dark chemistry. In smaller fires in the southeast United States, close to 40% of the OA is influenced by dark chemistry.

## Discussion

Dark chemical transformation of bbOA is currently not included in atmospheric models; however, our results here show that much of the bbOA in the ambient atmosphere during periods of moderate to low photochemical activity can be substantially influenced by dark NO_3_ aging. Our laboratory results indicate that not only does bbOA exposed to NO_2_ and O_3_ rapidly age in the dark, but the produced SOA is chemically similar to observed OOA factors in wintertime urban areas affected by residential BB. High RH conditions further promote the oxidation state and amount of OOA from BB. Additionally, dark aging in the presence of NO_2_ and O_3_ produces substantial amounts of nitrate aerosol, which can contribute to strong pollution episodes seen worldwide (e.g., wintertime China). The rapid oxidation and considerable amount of bbOOA generated reshapes the conceptual model of oxidation of bbOA, and further elevates the importance of BB as a source of OOA worldwide. Given that model results indicate that dark aging influences nearly all bbOA, it is essential to understand the implications of this neglected oxidation pathway on the atmospheric load of OA, and the resulting impact on climate and health.

We note several key topics for additional work. First, the full chemical mechanism for NO_3_ oxidation of bbOA is unclear. Consistent with previous studies (28), we observe the initial reactions of NO_3_ with phenol, cresol, and furanoic compounds (*SI Appendix*, Fig. S4); however, the subsequent steps are less well constrained. A second topic is the process (or processes) by which the bbOOA is formed. The large enhancement in secondary aerosol suggests that homogenous gas-phase oxidation and subsequent condensation of lower-volatility vapors is probably the dominant process. However, we cannot rule out the possible role of heterogeneous oxidation processes. Finally, the chemical scheme used in the chemical transport model to simulate this dark NO_3_ oxidation is clearly oversimplified and requires improvement.

## Materials and Methods

### Environmental Chamber Facility and Instrumentation.

Experiments of chemical aging of BB emissions took place at the Foundation for Research & Technology-Hellas (FORTH) atmospheric simulation chamber and combustion chamber facilities at the Center for the Study of Air Quality & Climate Change (http://cstacc.iceht.forth.gr). Details of the chamber facility have been discussed previously (40). Briefly, this facility consists of a 10-m^3^ Teflon reactor inside a 30-m^3^ temperature- and light-controlled room. The chamber room has aluminum-coated walls and is equipped with series of black light lamps that emit in the wavelength range between 300 and 450 nm. Combustion emissions were transferred into the environmental chamber from a combustion chamber through a dilution system. The combustion facility is located in a separate room directly underneath the environmental chamber. Commercially available fuel (olive tree wood) and a wood stove were used to generate primary BB emissions. The fuel and heating stove were bought locally and represent typical fuel and heating devices used in this region.

Aerosol and gas-phase species were monitored continuously through a number of online instruments during each experiment. An HR-ToF-AMS (Aerodyne Research Inc.) measured the nonrefractory particulate matter with diameters less than 1 micron (PM_1_) aerosol (41). A scanning mobility particle sizer (classifier model 3080, DMA model 3081, CPC model 3787, TSI) measured the particle number size distribution. A multiple-angle absorption photometer (Thermo Scientific Inc.) was used for the measurement of the PM_1_ particulate black carbon. VOCs were measured by a proton-transfer-reaction mass spectrometer (PTR-MS, Ionicon Analytik). Finally, a series of gas monitors was used for the measurement of the mixing ratios of the nitrogen oxides (NO and NO_2_), ozone (O_3_), carbon monoxide (CO), and carbon dioxide (CO_2_) (Teledyne models T201, 400E, 300E, and T360, respectively). Further online instrument descriptions and operating conditions are discussed in Kaltsonoudis et al. (40).

### Experimental Procedure.

*SI Appendix*, Table S1 describes the 10 experiments presented in this study. Experiments 1 and 2 are reference experiments in dark conditions with no added oxidants (dark reference) or with UV lights (UV reference) to compare the magnitude of aging under dark conditions. Experiments 3 to 6 and 10 are aged in dark and dry (RH less than 10%) conditions with differing amounts of injected oxidant concentrations. Experiments 7 to 9 are aged in dark conditions under high RH (50 to 80%).

All experiments followed a similar procedure, with some differences in design to explore the sensitivity of chemical aging to RH or oxidant concentrations. In general, the BB emissions were injected into the chamber via a dilution system over a roughly 30- to 40-min period. The emissions were then left in dark conditions for approximately 2 h to allow for sufficient mixing and characterization of the fresh emissions and conditions of the environmental chamber (e.g., particle wall loss rates). After this initial characterization period, oxidation was initiated through injection of O_3_ (for the dark aging experiments) or turning on UV lights (for the UV reference experiment). The initiation of oxidation is defined as time 0. The BB emissions were sampled continuously by the online suite of instrumentation for an additional 3 h to 10 h (depending on the volume of air remaining in the chamber). Here, aging results are presented for the first 3 h for consistency, as this period contains the majority of the enhancement in aging metrics. The concentrations of OH radical were estimated by monitoring the change in isotopically labeled butanol (1-butanol-d9, Sigma) measured by the PTR-MS at *m*/*z* 66 following Barmet et al. (42). We find no evidence for OH exposure in any of the dark aging experiments (*SI Appendix*, Fig. S11).

### Data Analysis and Calculation of Enhancement Factors.

Measurements from the HR-ToF-AMS were analyzed using the AMS software toolkits, SeQUential Igor data RetRiEvaL (SQUIRREL) v1.57 and the Peak Integration by Key Analysis (PIKA) v1.16. The oxygen-to-carbon ratio was calculated following the approach of Canagaratna et al. (43). Organic nitrates are calculated from the measured ratio of NO_2_^+^ to NO^+^ by the AMS, based on the approach discussed in Kiendler-Scharr et al. (27) and Farmer et al. (44).

Enhancement factors were calculated as the ratio of a value over the entire experiment relative to an initial value defined as the average of the value from times of hour −1 to −0.5. Specifically, the enhancement factors are equal to C(t)/C0, where C is the O:C ratio (Fig. 2*A*), the ratio of *f*_44_/*f*_60_ (Fig. 2*B*), the ratio of OA to sulfate (Fig. 2*C*), or the ratio of aerosol nitrate to sulfate (Fig. 2*D*). Normalizing to the sulfate concentration in Fig. 2 *C* and *D* allows the calculation to be independent of corrections to collection efficiency and particle wall loss. Different averaging time windows were tested for the initial time, and the results were found not to be sensitive to the initial time.

### Calculation of Laboratory Fresh and Aged bbOA.

To calculate the fresh and aged OA mass spectra in the laboratory environmental chamber experiments, we follow the mass balance approach outlined in Jorga et al. (45). Briefly, this method separates an initial (i.e., fresh) and produced (i.e., aged) OA factor based on the assumption that particle wall loss and SOA formation are the dominant processes occurring in the chamber. The fresh OA factor is defined as the OA prior to the initiation of oxidation. The time series of this factor is modeled assuming this factor decays by a first-order loss rate with the observed particle wall loss rate constant of this chamber. *SI Appendix*, Fig. S12 shows the extrapolation of the initial OA factor for the dark aging experiment under dry conditions shown in Fig. 3 (experiment 3). The particle wall loss rate constant for this chamber was measured to be 0.06 h^−1^ (uncertainty range: 0.042 h^−1^). The mass spectrum for this initial factor evolves assuming that OA at each *m/z* decays at the same rate. The produced OA is the difference between the total measured OA and the initial OA factor. A key limitation of this method is that it does not account for additional processes in the chamber, notably, evaporation of particles and subsequent vapor loss to walls or heterogeneous reactions on the particle surface. In experiment 3 (plotted in *SI Appendix*, Fig. S12 and Fig. 3), the initial OA in the chamber follows the observed particle wall loss decay rate for the time period prior to initiation of oxidation, suggesting evaporation and loss to walls is a minor process on the time scale of this experiment.

### Comparison to Ambient Observations.

We compare the bbOA and produced bbOOA from the laboratory experiments to ambient observations from published studies (*SI Appendix*, Tables S2 and S3). These sites were chosen due to the influence of residential BB in an urban environment. We use the HR AMS mass spectra for all sites with the exception of Paris, France, Po Valley, Italy, and Fresno, CA, where only the unit mass resolution AMS mass spectra were available. To quantify the degree of similarity between two spectra, we calculate the angle “theta” between the spectra (treating the spectra as vectors) defined by the inner product (46).

### Chemical Transport Model Setup.

Model simulations were performed on the chemical transport model, Particulate Matter Comprehensive Air quality Model with extensions (PMCAMx), over a regional domain centered on the United States in autumn (October 5 to November 15, 2011). A horizontal resolution of 36 × 33.6 km and vertical resolution of 25 layers ranging from the surface to 19 km was used in this application. Transport in the model is driven by meteorology fields generated by the Weather Research and Forecast Model version 3.3.1. This version of PMCAMx was extended to treat bbOA explicitly, a configuration known as PMCAMx-SR (47). This version of PMCAMx-SR uses the same modules of PMCAMx, but tracks bbOA separately from other OA sources. The volatility of OA is modeled using the volatility basis set approach (48). We use the May et al. (49) volatility distribution to describe the volatility of bbOA emissions and the Jathar et al. (50) emission factors for BB-IVOCs.

Oxidation in PMCAMx-SR is extended to include reactions of primary BB emissions with the NO_3_ radical. We assume that BB VOCs species react with the NO_3_ radical to produce gas-phase species of the same volatility as the reactant. We use a rate constant for this reaction of k_no3_ = 1.4 × 10^−11^ cm^3^⋅molec^−1^⋅s^−1^, similar to reactions of cresol with the NO_3_ radical (51). We further assume that the secondary gas-phase products of the reaction with the NO_3_ radical can react with the OH radical to form gas-phase products with a reduction of one volatility bin.

## Supplementary Material

Supplementary File

## Data Availability

Data have been deposited in EUROCHAMP-2020 (Integration of European Simulation Chambers for Investigating Atmospheric Processes–towards 2020 and beyond) public repository (https://data.eurochamp.org/). Data can be obtained from https://data.eurochamp.org/data-access/chamber-experiments by selecting search terms 'Biomass Burning' for Compounds and 'FORTH' for Institute.

## References

[1] Pandis S. N., Urban particulate matter pollution: A tale of five cities. Faraday Discuss. 189, 277–290 (2016). 27310460 10.1039/c5fd00212e

[2] Zhang Q., Ubiquity and dominance of oxygenated species in organic aerosols in anthropogenically-influenced Northern Hemisphere midlatitudes. Geophys. Res. Lett. 34, L13801 (2007).

[3] Zhang Q., Worsnop D. R., Canagaratna M. R., Jimenez J. L., Hydrocarbon-like and oxygenated organic aerosols in Pittsburgh: Insights into sources and processes of organic aerosols. Atmos. Chem. Phys. 5, 3289–3311 (2005).

[4] Jimenez J. L., Evolution of organic aerosols in the atmosphere. Science 326, 1525–1529 (2009). 20007897 10.1126/science.1180353

[5] Kanakidou M., Organic aerosol and global climate modelling: A review. Atmos. Chem. Phys. 5, 1053–1123 (2005).

[6] Robinson A. L., Rethinking organic aerosols: Semivolatile emissions and photochemical aging. Science 315, 1259–1262 (2007). 17332409 10.1126/science.1133061

[7] Jacob D. J., Introduction to Atmospheric Chemistry (Princeton University Press, 1999).

[8] Pai S. J., An evaluation of global organic aerosol schemes using airborne observations. Atmos. Chem. Phys. Discuss. 2019, 1–39 (2019).

[9] Fountoukis C., Simulating the formation of carbonaceous aerosol in a European Megacity (Paris) during the MEGAPOLI summer and winter campaigns. Atmos. Chem. Phys. 16, 3727–3741 (2016).

[10] Tsimpidi A. P., Karydis V. A., Pandis S. N., Lelieveld J., Global combustion sources of organic aerosols: Model comparison with 84 AMS factor-analysis data sets. Atmos. Chem. Phys. 16, 8939–8962 (2016).

[11] Florou K., The contribution of wood burning and other pollution sources to wintertime organic aerosol levels in two Greek cities. Atmos. Chem. Phys. 17, 3145–3163 (2017).

[12] O’Dell K., Ford B., Fischer E. V., Pierce J. R., Contribution of wildland-fire smoke to US PM_2.5_ and its influence on recent trends. Environ. Sci. Technol. 53, 1797–1804 (2019). 30681842 10.1021/acs.est.8b05430

[13] Westerling A. L., Hidalgo H. G., Cayan D. R., Swetnam T. W., Warming and earlier spring increase Western U.S. Forest Wildfire Activity. Science 313, 940–943 (2006). 16825536 10.1126/science.1128834

[14] Ford B., Future fire impacts on smoke concentrations, visibility, and health in the contiguous United States. Geohealth 2, 229–247 (2018). 32159016 10.1029/2018GH000144 PMC7038896

[15] Fine P. M., Cass G. R., Simoneit B. R. T., Organic compounds in biomass smoke from residential wood combustion: Emissions characterization at a continental scale. J. Geophys. Res. Atmos. 107, ICC 11-1–ICC 11-9 (2002).

[16] Koss A. R., Non-methane organic gas emissions from biomass burning: Identification, quantification, and emission factors from PTR-ToF during the FIREX 2016 laboratory experiment. Atmos. Chem. Phys. 18, 3299–3319 (2018).

[17] Hennigan C. J., Chemical and physical transformations of organic aerosol from the photo-oxidation of open biomass burning emissions in an environmental chamber. Atmos. Chem. Phys. 11, 7669–7686 (2011).

[18] Hodshire A. L., Aging effects on biomass burning aerosol mass and composition: A critical review of field and laboratory studies. Environ. Sci. Technol. 53, 10007–10022 (2019). 31365241 10.1021/acs.est.9b02588

[19] Ng N. L., Nitrate radicals and biogenic volatile organic compounds: Oxidation, mechanisms, and organic aerosol. Atmos. Chem. Phys. 17, 2103–2162 (2017). 30147712 10.5194/acp-17-2103-2017 PMC6104845

[20] Brown S. S., Stark H., Ciciora S. J., Ravishankara A. R., In-situ measurement of atmospheric NO3 and N2O5 via cavity ring-down spectroscopy. Geophys. Res. Lett. 28, 3227–3230 (2001).

[21] Benton A. K., Night-time chemistry above London: Measurements of NO_3_ and N_2_O_5_ from the BT Tower. Atmos. Chem. Phys. 10, 9781–9795 (2010).

[22] Rollins A. W., , Evidence for NOx control over nighttime SOA formation. Science 337, 1210–1212 (2012). 22955831 10.1126/science.1221520

[23] Ng N. L., Secondary organic aerosol (SOA) formation from reaction of isoprene with nitrate radicals (NOx). Atmos. Chem. Phys. 8, 4117–4140 (2008).

[24] Brown S. S., Biogenic VOC oxidation and organic aerosol formation in an urban nocturnal boundary layer: Aircraft vertical profiles in Houston, TX. Atmos. Chem. Phys. 13, 11317–11337 (2013).

[25] Brown S. S., Effects of NO_x_ control and plume mixing on nighttime chemical processing of plumes from coal‐fired power plants. J. Geophys. Res. Atmos. 117, D07304 (2012).

[26] Brown S. S., Nocturnal isoprene oxidation over the Northeast United States in summer and its impact on reactive nitrogen partitioning and secondary organic aerosol. Atmos. Chem. Phys. 9, 3027–3042 (2009).

[27] Kiendler-Scharr A., Ubiquity of organic nitrates from nighttime chemistry in the European submicron aerosol. Geophys. Res. Lett. 43, 7735–7744 (2016).

[28] Hartikainen A., Volatile organic compounds from logwood combustion: Emissions and transformation under dark and photochemical aging conditions in a smog chamber. Environ. Sci. Technol. 52, 4979–4988 (2018). 29517225 10.1021/acs.est.7b06269

[29] Decker Z. C. J., Nighttime chemical transformation in biomass burning plumes: A box model analysis initialized with aircraft observations. Environ. Sci. Technol. 53, 2529–2538 (2019). 30698424 10.1021/acs.est.8b05359

[30] Tiitta P., Transformation of logwood combustion emissions in a smog chamber: Formationof secondary organic aerosol and changes in the primary organic aerosol upon daytime and nighttime aging. Atmos. Chem. Phys. 16, 13251–13269 (2016).

[31] Li C., formation of secondary Brown carbon in biomass burning aerosol Proxies through NO_3_ radical reactions. Environ. Sci. Technol. 54, 1395–1405 (2020). 31730747 10.1021/acs.est.9b05641

[32] Alfarra M. R., Identification of the mass spectral signature of organic aerosols from wood burning emissions. Environ. Sci. Technol. 41, 5770–5777 (2007). 17874785 10.1021/es062289b

[33] Ng N. L., Organic aerosol components observed in Northern Hemispheric datasets from aerosol mass spectrometry. Atmos. Chem. Phys. 10, 4625–4641 (2010).

[34] Gilardoni S., Direct observation of aqueous secondary organic aerosol from biomass-burning emissions. Proc. Natl. Acad. Sci. U.S.A. 113, 10013–10018 (2016). 27551086 10.1073/pnas.1602212113 PMC5018753

[35] Mohr C., Identification and quantification of organic aerosol from cooking and other sources in Barcelona using aerosol mass spectrometer data. Atmos. Chem. Phys. 12, 1649–1665 (2012).

[36] Saarikoski S., Chemical characterization of springtime submicrometer aerosol in Po Valley, Italy. Atmos. Chem. Phys. 12, 8401–8421 (2012).

[37] Crippa M., Wintertime aerosol chemical composition and source apportionment of the organic fraction in the metropolitan area of Paris. Atmos. Chem. Phys. 13, 961–981 (2013).

[38] Ge X., Setyan A., Sun Y., Zhang Q., Primary and secondary organic aerosols in Fresno, California during wintertime: Results from high resolution aerosol mass spectrometry. J. Geophys. Res. Atmos. 117, D19301 (2012).

[39] Chen C.-L., Organic aerosol particle chemical properties associated with residential burning and fog in wintertime San Joaquin Valley (Fresno) and with vehicle and firework emissions in summertime south coast air basin (Fontana). J. Geophys. Res. Atmos. 123, 10,707–710, 731 (2018).

[40] Kaltsonoudis C., Characterization of fresh and aged organic aerosol emissions from meat charbroiling. Atmos. Chem. Phys. 17, 7143–7155 (2017).

[41] DeCarlo P. F., Field-deployable, high-resolution, time-of-flight aerosol mass spectrometer. Anal. Chem. 78, 8281–8289 (2006). 17165817 10.1021/ac061249n

[42] Barmet P., OH clock determination by proton transfer reaction mass spectrometry at an environmental chamber. Atmos. Meas. Tech. 5, 647–656 (2012).

[43] Canagaratna M. R., Elemental ratio measurements of organic compounds using aerosol mass spectrometry: Characterization, improved calibration, and implications. Atmos. Chem. Phys. 15, 253–272 (2015).

[44] Farmer D. K., Response of an aerosol mass spectrometer to organonitrates and organosulfates and implications for atmospheric chemistry. Proc. Natl. Acad. Sci. U.S.A. 107, 6670–6675 (2010). 20194777 10.1073/pnas.0912340107 PMC2872396

[45] Jorga S. D., Kaltsonoudis C., Liangou A., Pandis S. N., Measurement of formation rates of secondary aerosol in the ambient urban atmosphere using a dual smog chamber system. Environ. Sci. Technol. 54, 1336–1343 (2020). 31869213 10.1021/acs.est.9b03479

[46] Kostenidou E., Lee B.-H., Engelhart G. J., Pierce J. R., Pandis S. N., Mass spectra deconvolution of low, medium, and high volatility biogenic secondary organic aerosol. Environ. Sci. Technol. 43, 4884–4889 (2009). 19673280 10.1021/es803676g

[47] Theodoritsi G. N., Pandis S. N., Simulation of the chemical evolution of biomass burning organic aerosol. Atmos. Chem. Phys. 19, 5403–5415 (2019).

[48] Donahue N. M., Epstein S. A., Pandis S. N., Robinson A. L., A two-dimensional volatility basis set: 1. Organic-aerosol mixing thermodynamics. Atmos. Chem. Phys. 11, 3303–3318 (2011).

[49] May A. A., Gas-particle partitioning of primary organic aerosol emissions: 3. Biomass burning. J. Geophys. Res. Atmos. 118, 11,327–11,338 (2013).

[50] Jathar S. H., Unspeciated organic emissions from combustion sources and their influence on the secondary organic aerosol budget in the United States. Proc. Natl. Acad. Sci. U.S.A. 111, 10473–10478 (2014). 25002466 10.1073/pnas.1323740111 PMC4115499

[51] ENVIRON, User’s Guide to the Comprehensive Air Quality Model with Extensions (CAMx) (ENVIRON Corporation, 2004).

