# Supplementary material for: Rapid dark aging of biomass burning as an overlooked source of oxidized organic aerosol
Source: Proc Natl Acad Sci U S A. 2020 Dec 14;117(52):33028–33. doi: 10.1073/pnas.2010365117 (PMC7776776; doi:10.1073/pnas.2010365117)
Supplement: Supplementary File [file pnas.2010365117.sapp.pdf]

SUPPLEMENTAL INFORMATION

Rapid dark aging of biomass burning as an overlooked source of oxidized organic aerosol

John K. Kodros[1], Dimitrios K. Papanastasiou [1,*], Marco Paglione[1,2], Mauro Masiol[1], Stefania Squizzato[1], Kalliopi Florou[1], Ksakousti Skyllakou[1], Christos Kaltsonoudis[1], Athanasios Nenes[1,3], Spyros N. Pandis[1,4]

[1]Institute of Chemical Engineering Sciences, ICE-HT, Patras, Greece

[2]Italian National Research Council - Institute of Atmospheric Sciences and Climate (CNR-ISAC), Bologna, Italy

[3]ENAC/IIE Swiss Federal Institute of Technology Lausanne (EPFL), Lausanne, Switzerland

[4]Department of Chemical Engineering, University of Patras, Patras, Greece

[*]now at: Buffalo Research Laboratories, Honeywell International Inc., 20 Peabody St., Buffalo NY 14210 USA

www.pnas.org/cgi/doi/10.1073/pnas.2010365117

Table S1. Experimental description and procedure. Concentrations of O_3 and NO_2 correspond to initial observed values after the O_3 injection (hour 0). Relative humidity corresponds to the value after the biomass burning injection. O:C, f_{44}/f_{60}, OA, and nitrate (aerosol) are expressed as relative enhancement factors (see Methods). Experiments in bold are used as representative experiments in the main and supplemental text.

**Experiment**	**O_3** [ppb]	**NO_2** [ppb]	**Dark/ Lights**	**RH**	**O:C**	**f_{44}/f_{60}**	**OA**	**Nitrate**
**Exp. 1**	-	-	Dark	10%	1.01	1.10	0.93	1.22
**Exp. 2**	-	-	Lights	10%	1.82	4.85	1.16	0.85
**Exp. 3**	81	88	Dark	10%	1.30	2.03	1.51	4.04
Exp. 4	37	35	Dark	10%	1.20	1.57	1.32	3.63
Exp. 5	88	43	Dark	10%	1.36	2.53	1.77	5.62
Exp. 6	44	88	Dark	10%	1.25	2.30	1.70	2.57
Exp. 7	90	95	Dark	50%	1.46	4.85	1.64	4.00
**Exp. 8**	90	84	Dark	75%	1.40	4.15	1.15	1.96
Exp. 9	83	92	Dark	70%	1.48	6.05	1.33	2.81
Exp. 10	43	-	Dark	10%	1.19	1.66	1.22	1.83

Table S2. Comparisons (quantified by the theta angle) to ambient AMS OA factors from previous studies to the produced bbOOA in the dark-dry and dark-RH laboratory experiments from this study.

Reference	Location	Factor	Dark-Dry Produced OOA [degrees]	Dark-RH Produced OOA [degrees]
Florou et al. (1)	Patras, Greece	OOA [HR]	13	11
Florou et al. (1)	Athens, Greece	OOA [HR]	17	19
Gilardoni et al. (2)	Bologna, Italy	OOA [HR]	15	20
Crippa et al. (3)	Paris, France	LV-OOA [UMR]	14	9
Crippa et al. (3)	Paris, France	OOA2 [UMR]	20	28
Saarikoski et al. (4)	Po Valley, Italy	OOAa [UMR]	16	10
Saarikoski et al. (4)	Po Valley, Italy	OOAb [UMR]	8	9
Ge et al. (5)	Fresno, USA	OOA [UMR]	17	26
Mohr et al. (6)	Barcelona, Spain	LVOOA [HR]	14	9
Chen et al. (7)	Fresno, USA	NOOA [HR]	31	32

Table S3: Comparisons (quantified by the theta angle) to ambient AMS OA factors from previous studies to the produced bbOA in the dark-dry and dark-RH laboratory experiments from this study.

Reference	Location	Factor	Dark-Dry bbOA	Dark-RH bbOA
Florou et al. (1)	Patras, Greece	BBOA [HR]	20	19
Florou et al. (1)	Athens, Greece	BBOA [HR]	19	23
Gilardoni et al. (2)	Bologna, Italy	BBOA [HR]	27	32
Crippa et al. (3)	Paris, France	BBOA [UMR]	23	29
Saarikoski et al. (4)	Po Valley, Italy	BBOA [UMR]	30	33
Ge et al. (5)	Fresno, USA	BBOA [UMR]	16	13
Mohr et al. (6)	Barcelona, Spain	BBOA [HR]	36	36
Chen et al. (7)	Fresno, USA	BBOA [HR]	27	27

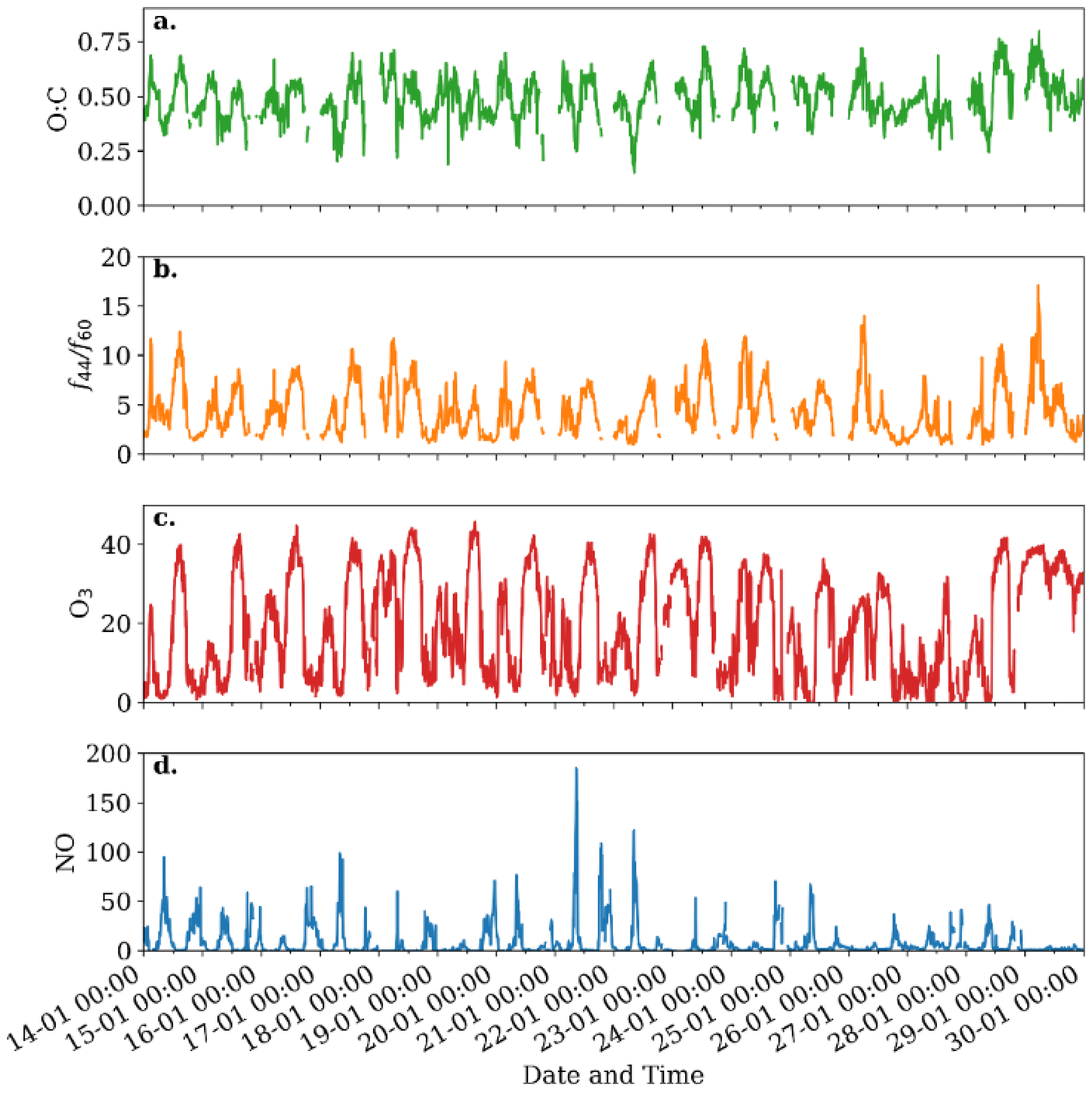

Figure S1. Time series from 16-days of ambient observations taken in Patras, Greece between January 14-30 2020 of **(a)** O:C and **(b)** f_{44}/f_{60} measured with the HR-ToF-AMS as well as **(c)** O_3 and **(d)** NO.

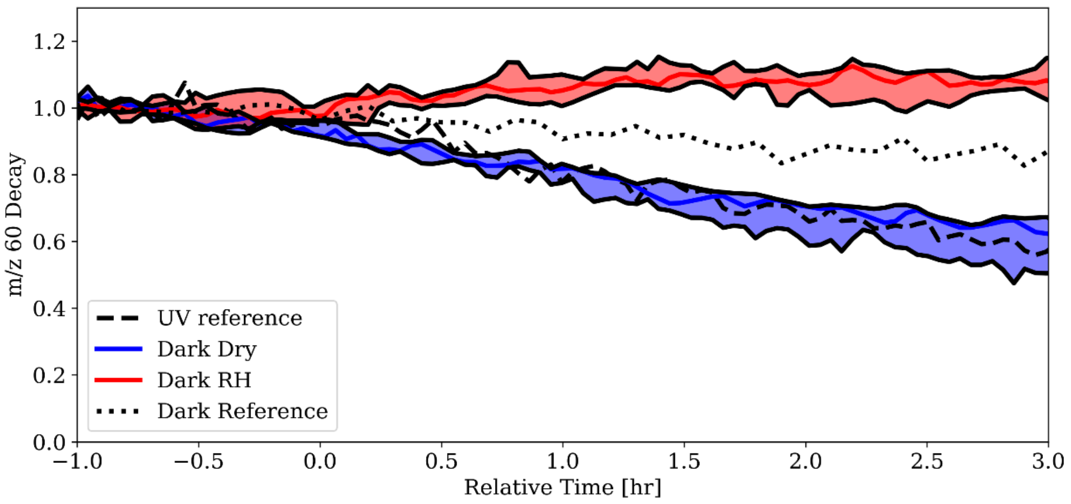

Figure S2. The decay of AMS-measured OA signal at *m*/*z* 60 (normalized to SO_4 to account for particle wall loss and collection efficiency corrections). The OA signal at *m*/*z* 60 (representative of levoglucosan and other sugars) decays in the dark-aging experiments (Exp. 7-9) under high RH conditions a similar amount as under UV conditions (Exp. 2); however, under dark dry conditions (Exp. 3-6), the signal at *m*/*z* 60 increases by approximately 10%.

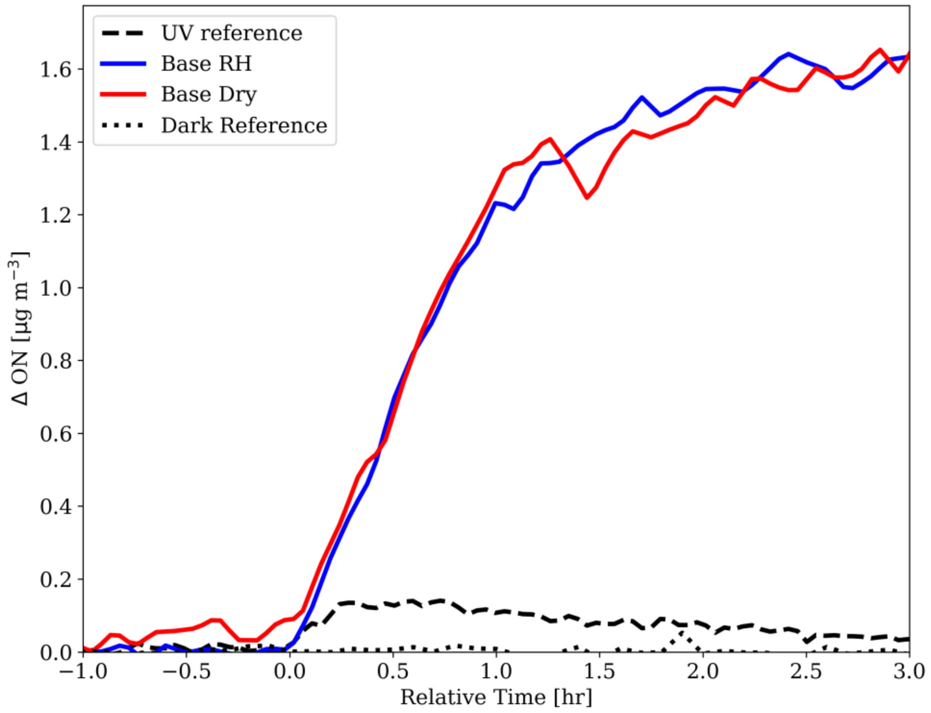

Figure S3. The change in organic nitrate mass concentration from the conditions prior to the initiation of oxidation for representative experiments under UV light conditions (*UV reference*, Exp. 2), dark and dry conditions (Exp. 3), dark and high RH conditions (Exp. 8), and dark with no oxidants (*dark reference*, Exp. 1).

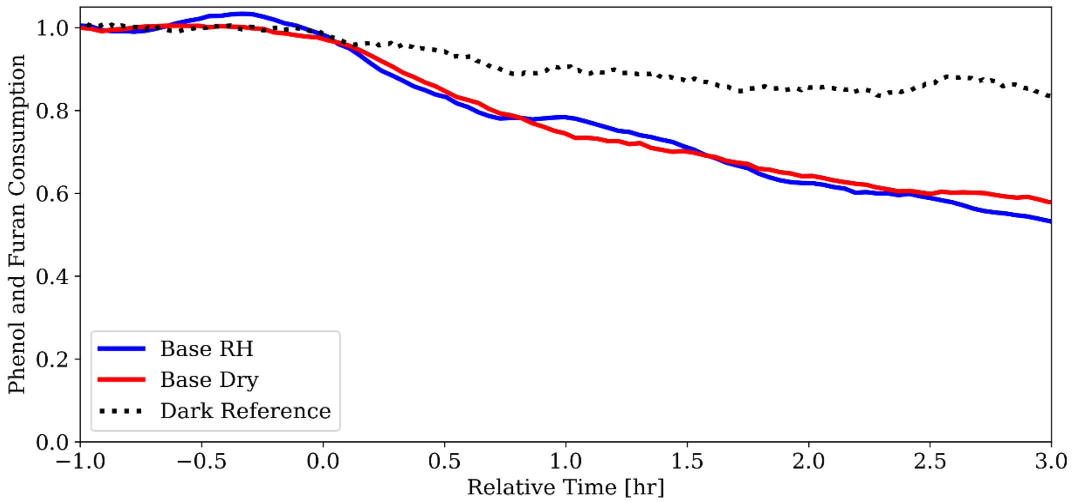

Figure S4. The decay of the summed phenolic and furanoic compounds relative to initial conditions prior to the initiation of oxidation measured from the PTR-MS in representative dark aging experiments under dry (Exp. 3) and high RH conditions (Exp. 8) as well as the *dark reference* experiment with no added oxidations (Exp. 1).

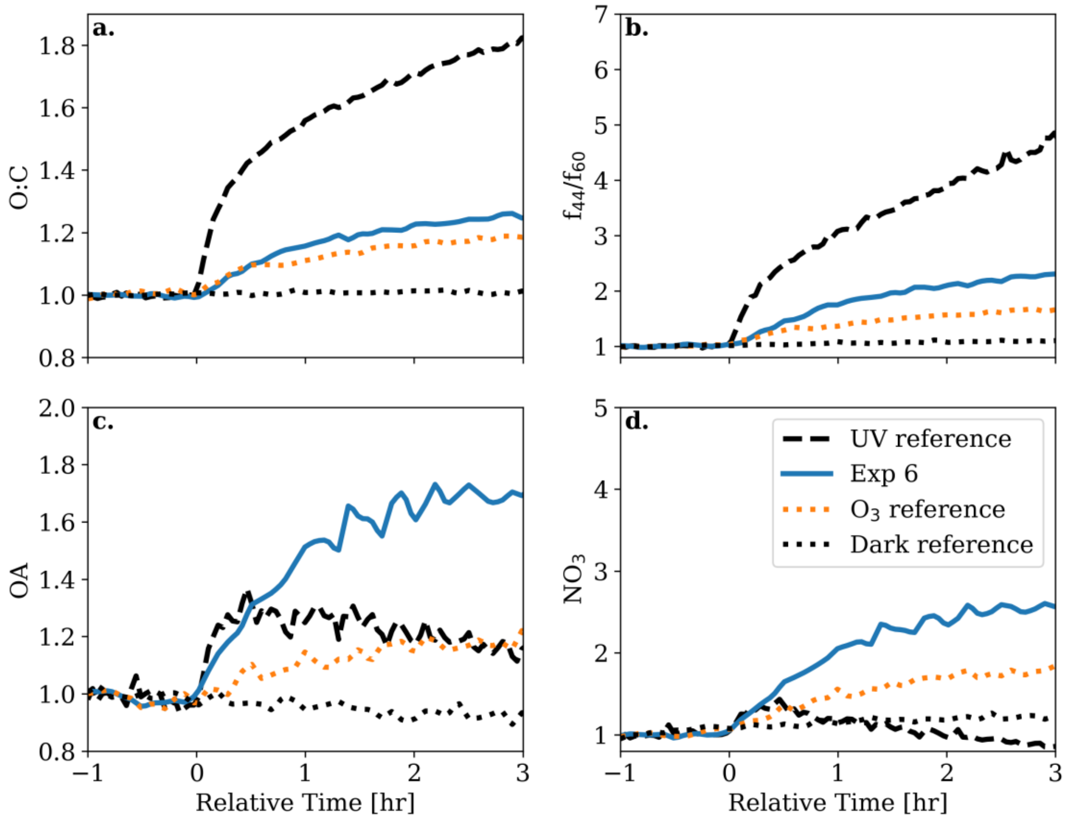

Figure S5. The enhancement in **(a)** O:C ratio, **(b)** f_{44}/f_{60}, **(c)** OA, **(d)** NO_3 aerosol (as in Figure 2 in the main text) for a dark aging experiment with only an injection of O_3 (Exp. 10) and an experiment with a similar injection of O_3 as well as NO_2 (Exp. 6). We note, however, that the BB combustion itself emitted 12 ppb of NO_x ($NO+NO_2$) in the O_3 reference (Exp. 10) experiment, and so this cannot be considered only containing O_3.

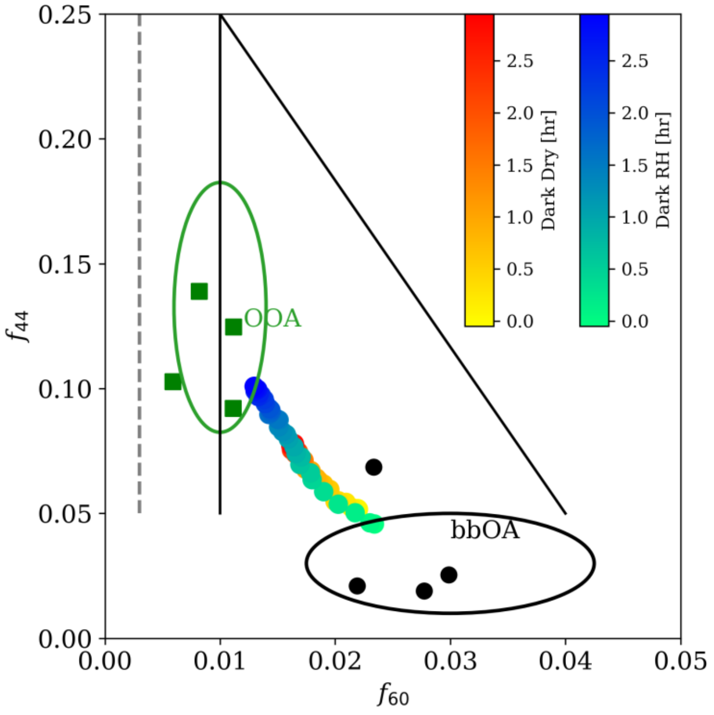

Figure S6. Evolution of HR-ToF-AMS measured OA through f_{60} versus f_{44} space for a representative dark aging experiment under dry (Exp. 3) and high RH (Exp. 8) conditions along with ambient bbOA (black circles) and OOA (green squares) factors.

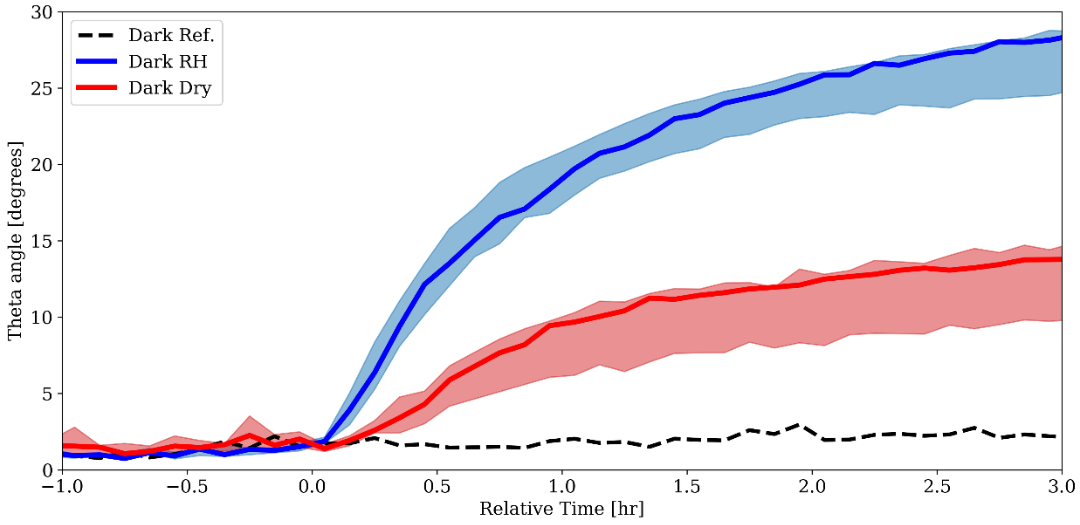

Figure S7. The evolution of the theta angle between the OA spectrum during the experiment relative to the average spectrum prior to the initiation of oxidation.

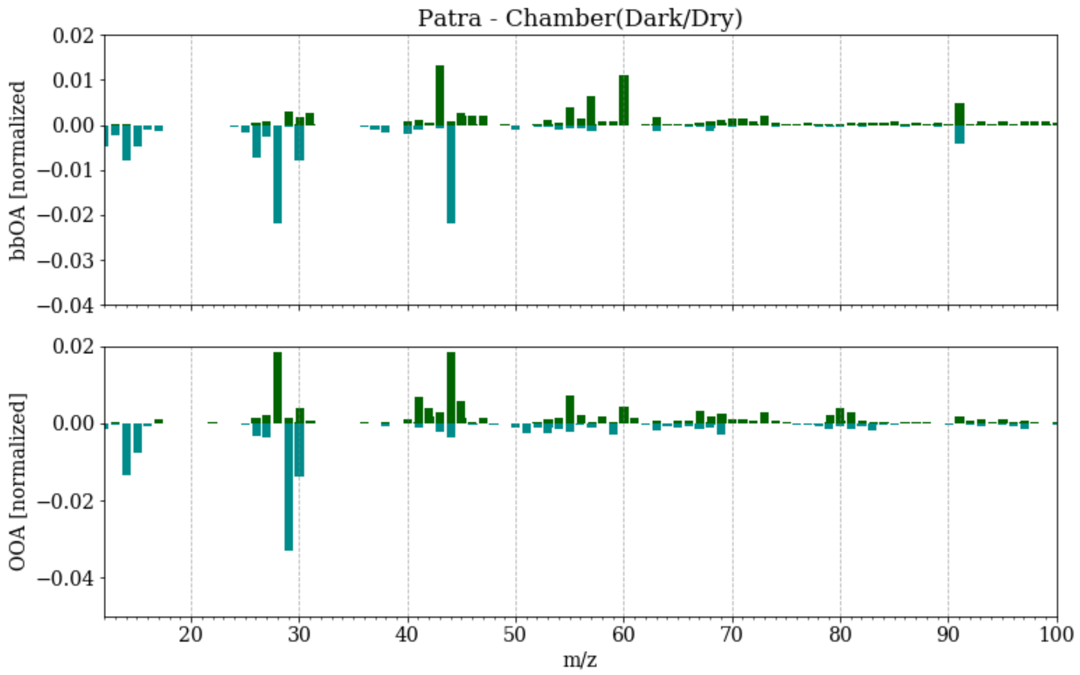

Figure S8. Difference in normalized spectra between the dark-dry chamber experiment and the ambient observations of bbOA and OOA factors in Patras, Greece.

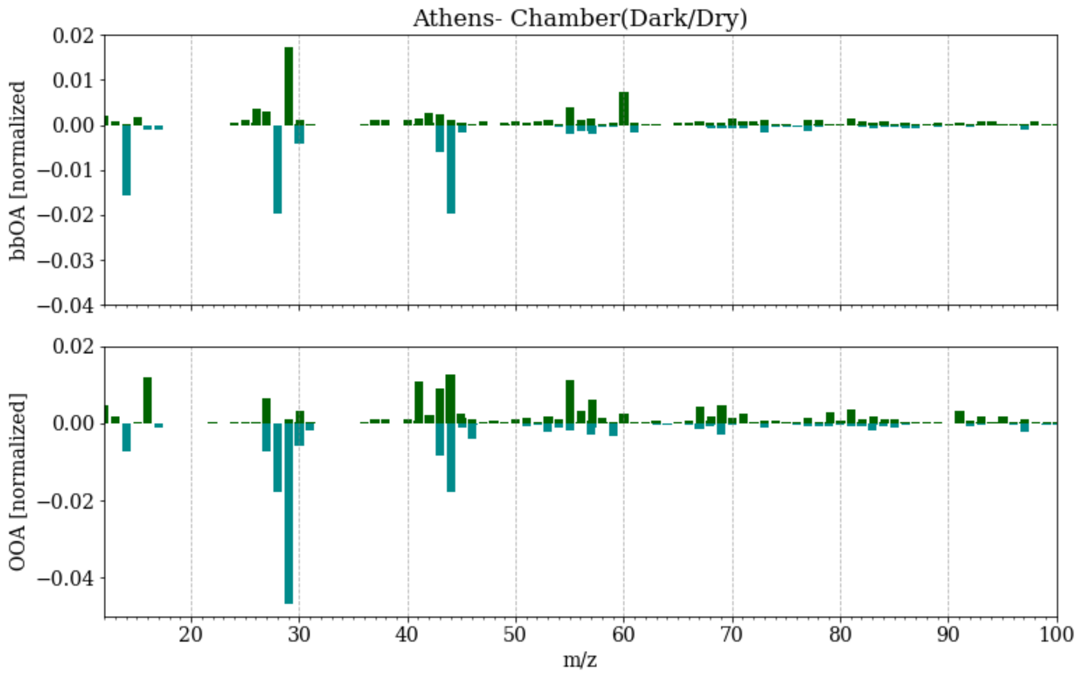

Figure S9. Difference in normalized spectra between the dark-dry chamber experiment and the ambient observations of bbOA and OOA factors in Athens, Greece.

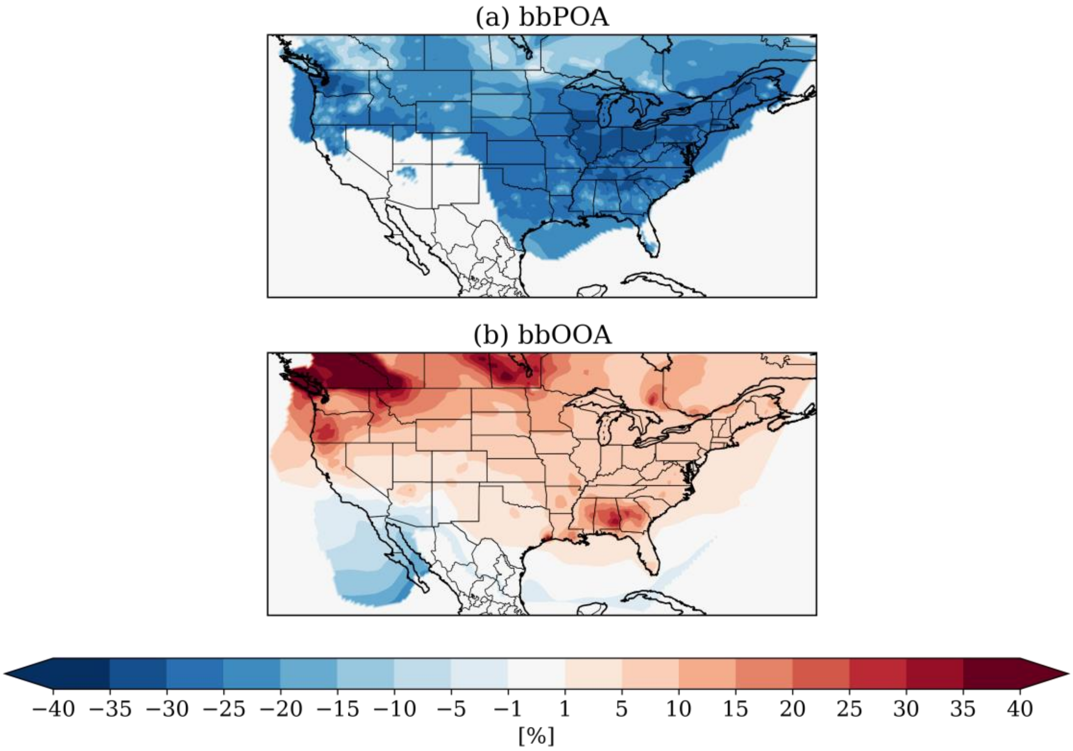

Figure S10. The percent difference in simulated **(a)** bbPOA and **(b)** bbOOA between model simulations with NO_3 oxidation of BB-VOCs relative to simulations with only OH radical oxidation.

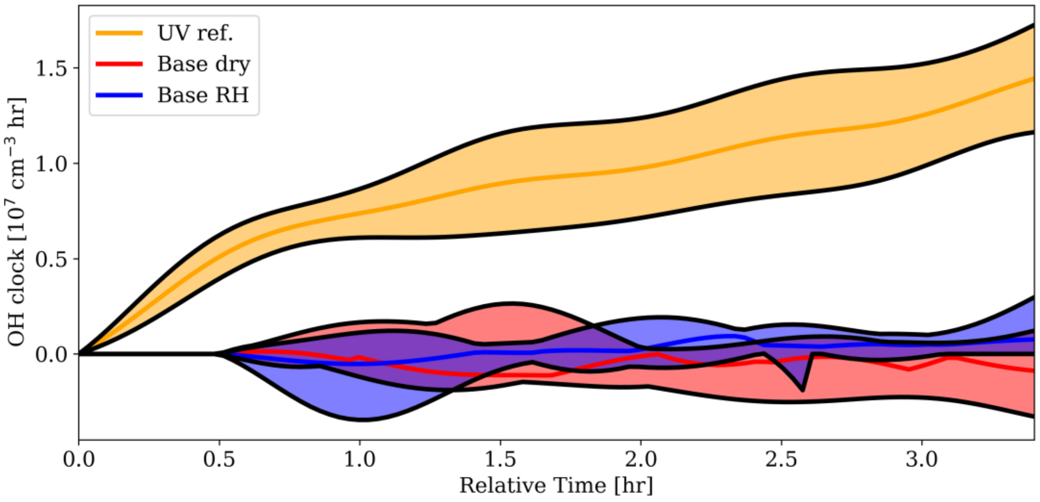

Figure S11. Integrated OH exposure during the experiments with and without lights.

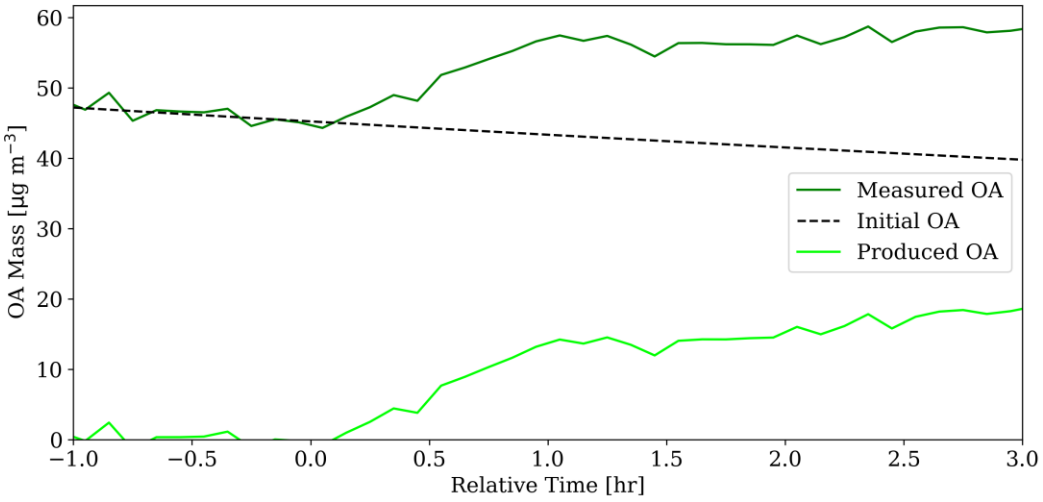

Figure S12. Time series of measured chamber OA from the representative dark aging experiment under dry conditions (Exp. 3) split into an initial (i.e. fresh) and produced (i.e. aged) OA factor using the mass-balance approach of Jorga et al. (2019).

References

1. K. Florou, *et al.*, The contribution of wood burning and other pollution sources to wintertime organic aerosol levels in two Greek cities. *Atmos. Chem. Phys.* **17**, 3145–3163 (2017).
2. S. Gilardoni, *et al.*, Direct observation of aqueous secondary organic aerosol from biomass-burning emissions. *Proc. Natl. Acad. Sci.* **113**, 10013 LP – 10018 (2016).
3. M. Crippa, *et al.*, Wintertime aerosol chemical composition and source apportionment of the organic fraction in the metropolitan area of Paris. *Atmos. Chem. Phys.* **13**, 961–981 (2013).
4. S. Saarikoski, *et al.*, Chemical characterization of springtime submicrometer aerosol in Po Valley, Italy. *Atmos. Chem. Phys.* **12**, 8401–8421 (2012).
5. X. Ge, A. Setyan, Y. Sun, Q. Zhang, Primary and secondary organic aerosols in Fresno, California during wintertime: Results from high resolution aerosol mass spectrometry. *J. Geophys. Res. Atmos.* **117** (2012).
6. C. Mohr, *et al.*, Identification and quantification of organic aerosol from cooking and other sources in Barcelona using aerosol mass spectrometer data. *Atmos. Chem. Phys.* **12**, 1649–1665 (2012).
7. C.-L. Chen, *et al.*, Organic Aerosol Particle Chemical Properties Associated With Residential Burning and Fog in Wintertime San Joaquin Valley (Fresno) and With Vehicle and Firework Emissions in Summertime South Coast Air Basin (Fontana). *J. Geophys. Res. Atmos.* **123**, 10,707-710,731 (2018).
